# Additive Manufacturing Techniques for the Reconstruction of 3D Fetal Faces

**DOI:** 10.1155/2017/9701762

**Published:** 2017-12-19

**Authors:** Domenico Speranza, Daniela Citro, Francesco Padula, Barbara Motyl, Federica Marcolin, Michele Calì, Massimo Martorelli

**Affiliations:** ^1^Department of Civil and Mechanical Engineering, University of Cassino and Southern Lazio, Cassino, Italy; ^2^Department of Prenatal Diagnosis, ALTAMEDICA, Fetal Maternal Medical Centre, Rome, Italy; ^3^Polytechnic Department of Engineering and Architecture, University of Udine, Udine, Italy; ^4^Department of Management and Production Engineering, Polytechnic of Turin, Turin, Italy; ^5^Department of Electric, Electronics and Computer Engineering, University of Catania, Catania, Italy; ^6^Department of Industrial Engineering, University of Naples Federico II, Naples, Italy

## Abstract

This paper deals with additive manufacturing techniques for the creation of 3D fetal face models starting from routine 3D ultrasound data. In particular, two distinct themes are addressed. First, a method for processing and building 3D models based on the use of medical image processing techniques is proposed. Second, the preliminary results of a questionnaire distributed to future parents consider the use of these reconstructions both from an emotional and an affective point of view. In particular, the study focuses on the enhancement of the perception of maternity or paternity and the improvement in the relationship between parents and physicians in case of fetal malformations, in particular facial or cleft lip diseases.

## 1. Introduction

Additive manufacturing (AM) technologies are widely used in different application fields. From industrial to cultural heritage applications and coupled with reverse engineering and CAD modelling techniques, AM technologies can close the so-called design loop [1–9].

The use of AM in medical applications is promising in terms of both usage and benefits. In the near future, this sector will especially benefit from AM techniques for the creation of prostheses and 3D models and also for direct printing of human organs [10–13].

On the other hand, medical imaging systems, designed to extract three-dimensional information from human body organs through noninvasive methods (as ultrasounds), are increasingly advanced and are taking on the central role, leading also to the affirmation of medical imaging techniques [14].

In the present study, the possibility to use AM techniques in the field of the direct fabrication of models extracted from routine ultrasound data and in particular of 3D baby face printing applications has been explored [15, 16]. Also, the use of 3D fetal printing is considered as a support for both physicians and parents. In fact, physicians may use 3D models reconstructed from ultrasound, CT, and/or MRI data sets, for the representation of specific pathologies that would prepare them for dealing with different clinical scenarios of the future newborn [17–19], while parents can get ready to receive their newborn more consciously.

Menozzi et al. [20, 21] reported that 3D and 4D fetal visualization allows parents to start a useful emotional relationship with their newborn child. The view of the baby's first picture (2D or 3D) is one of the most memorable moments in the life of soon-to-be parents. It is the time pregnancy becomes real, and it is finally possible to admit that a human being is growing within the mother's body [22].

Recent studies [17–19, 23, 24] have shown that 3D modeling of ultrasound, MRI, and CT imaging can help physicians in prenatal screening of complex fetal anatomies and/or pathologies, for example, airway disorders, spinal or neurological injuries, and cleft lip [25, 26], and it can also help mothers in reducing the stress and anxiety of “not knowing well” possible deformations [27]. Pregnancy also appears to be a period of strong change for men, although it is slower and more indirect than for women. While waiting for the birth of his child, the man is engaged in the reelaboration of a new image of himself as a father and in a readaptation of his mental representations made during childhood [28]. Unlike women, men waiting for the birth of a child are not directly involved in the physical changes of the body during pregnancy. This means that the contact with the newborn, especially at the beginning and during pregnancy, is mediated by the changes in the body of the mother [29]. The “reality tests” of the child's existence, such as the pregnancy test, the first ultrasound, and the perception of fetal movements, play an important role in the transition to paternity and may favor the father's participation in pregnancy, allowing him to perceive the presence of the newborn more effectively and diminishing the sense of exclusion in the relationship with the partner [30].

In this context, the emotional and aesthetic impact of the use of a real 3D printed model is a fundamental aspect to consider. It is therefore appropriate to consider 3D printing as the next step of 3D/4D ultrasound, exploring the degree of interest in the model, defining how to promote the transition to parenthood and, above all, paternity. In addition, it is fascinating to research how this type of model can be perceived by the parents themselves in terms of utility, or in relation to possible fetal malformations especially at the facial level.

First, the paper describes a method for the extraction and elaboration of routine prenatal ultrasound medical images for the reconstruction of 3D printable models of fetal faces. The use of this type of models enables a more accurate study of the baby's shape and an early detection of any malformations or pathologies. Second, it presents the preliminary results of a survey conducted in collaboration with the Altamedica Clinic (Rome, Italy) to examine whether there are any derived benefits from the use of 3D print models for future parents both concerning enhancement in parental experience and improvement in the relationship with physicians in case of pathologies and/or fetal malformations using only image data of routine ultrasound examinations. The paper is outlined as follows. After the introduction and a brief background on the benefits from 3D fetal printing for both physicians and parents, section two describes the used reconstruction method while section three reports the questionnaire, discussion, and results. Finally, conclusions are drawn and suggestions for future research are made.

## 2. 3D Fetal Face Reconstruction Method

This section describes the method used for the reconstruction of 3D CAD printable models from fetal ultrasound volumes. The main steps of the proposed reconstruction method are those typical of medical imaging, namely, filtering, segmentation, and extraction of the region of interest (ROI). The aim of the process is to create 3D models, starting from data available for each pregnancy and for emotional and affective purposes and also with recognizable facial features, from which it will be also possible to extract information useful for early diagnosis of fetal malformations. The proposed method involves the following steps: image acquisition; image processing (extraction of ROI and relative point clouds); point cloud elaboration and merging; 3D CAD model construction; and 3D printing of the model.

**Figure 1:**
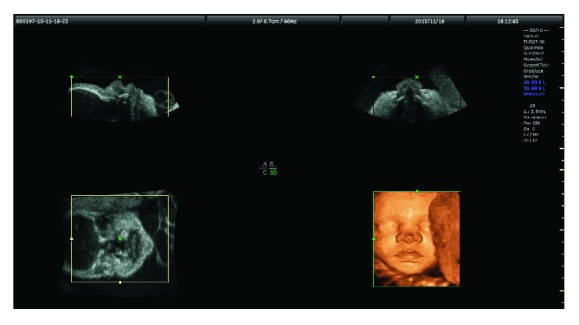
Screenshot of a 4D View application by GE Healthcare's Voluson.

The first phase, image acquisition, has been achieved by the use of GE Healthcare's Voluson ultrasound equipment during the routine checks with ultrasound for pregnancy [31]. The 4D View application manages and analyzes volumes from GE Healthcare's Voluson ultrasound data. GE software allows working offline to optimize, manipulate, and analyze data from the volumetric ultrasound, leaving the ultrasound physician to run more exams on more patients and improve the whole screening workflow. 4D View is fully integrated with patient folders, so the saved images and data can be accessed simultaneously and they are also transferable outside of the ultrasound system in DICOM format. Ultrasound images are converted to volumetric files (.VOO) that can only be processed by 4D View software. It is a powerful image management tool for enhanced patient throughput and department workflow in obstetrical ultrasound. It provides off-system 3D/4D volume manipulation and 2D image and clip review for a variety of Voluson ultrasound platforms (see Figure 1) and live reconstructions of sonogram imagery for “4D viewing” (3D viewing that animates in real-time). It is essentially based on a volume-rendering algorithm designed with known fetal and biological parameters to create realistic lighting conditions and scattering effects.

One of the main problems associated with this type of reconstruction procedure is due to the fact that 4D View files are proprietary, so they cannot be used in another software environment. The same is true if ultrasound instruments of other brands (e.g., Samsung Medison) are used. This way, it is impossible to use 3D data from 4D View to export a template (typically a STL file format) that is suitable for 3D printing.

A description of the proposed procedure, used for the construction of the 3D models presented in this study, is described as follows, emphasizing the most problematic steps and highlighting possible solutions using medical or commercial software applications.

**Figure 2:**
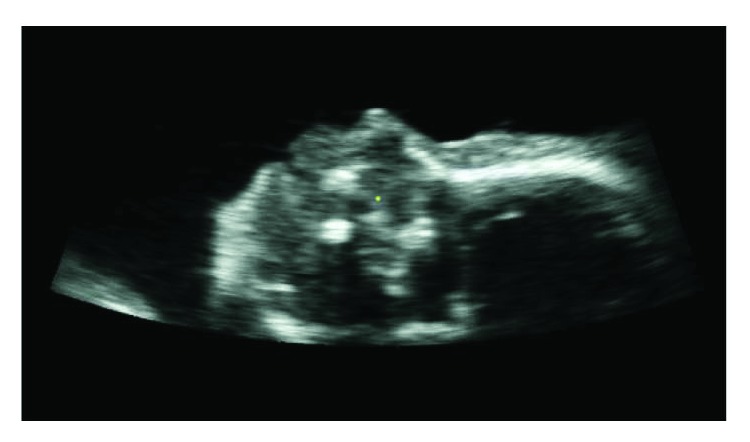
2D image or slice.

Using 4D View, after opening the file, it may be switch to the “full-screen multiple display mode,” choosing carefully one of the three section planes. During this operation, the magnifier parameter or magnifying command must be set to 1. Then progressive savings of the individual slice are performed, positioning them at the front of the entire data volume, until the content in the box disappears. For this study, a slice (Figure 2) consists of two-dimensional images representing an inner section of the object's volume, with a nonnull thickness ranging between 0.3 and 0.5 mm, which is the height of the considered voxels.

Between January and April 2017, about 40 fetal ultrasound models were rebuilt using data from the ultrasounds performed between the 20th and 22nd and between the 30th and 34th weeks of pregnancy.

One of the main disadvantages found in the use of 4D View software is that it is not possible to simultaneously save various sections that make up the whole volumetric file. In fact, it was necessary to repeat the saving operation up to 250 times, depending on the total number of slices that constituted the volume under elaboration.

A further disadvantage is represented by the lack of possibility to open a DICOM file, which would facilitate the slicing operations of the volume.

This way, the 2D image stack has to be exported using a graphic format (e.g., .jpeg, .bmp, .tiff) to be processed. In our case, the chosen software was Simpleware ScanIp, which allowed viewing, analyzing, quantifying segments, and exporting 3D images from different types of medical scans: MRI CT, microtomography, ultrasound, and so on.

**Figure 3:**
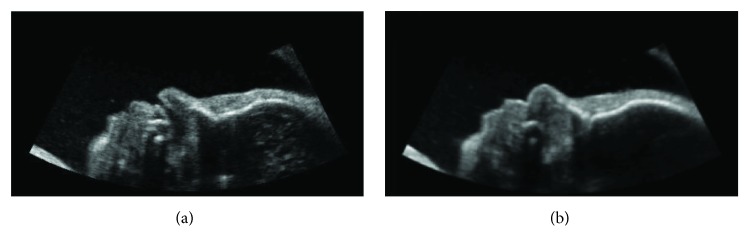
Smoothing: (a) slice before and (b) after application of the recursive Gaussian filter.

This way, after creating a new mask, it is advisable to apply smoothing algorithms to reduce the noise in the images. In these cases, noise consists of a random variation of brightness or color information. In fact, all of the digital image acquisition processes are characterized by noise-induced component of the capture system, which degrades the quality and therefore the information content of the data. It is important to reduce the noise as it may introduce a set of untruthful information inside digital images. After several experiments, the recursive Gaussian filter was used to blur the image and reduce the noise, with sigma value set to 1 [32].  Figure 3 highlights an example of the results obtained by the application of the recursive Gaussian filter to one single slice.

**Figure 4:**
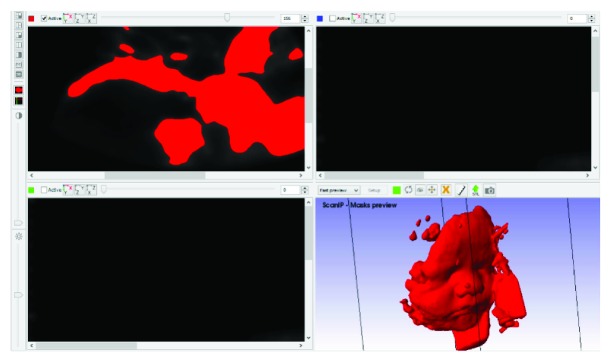
Steps of segmentation process.

Next step is the segmentation process (see Figure 4). In this phase, the digital image is divided into one or more pixel regions. The objective of the segmentation is to isolate or highlight certain ROI which have some specific characteristics or identifying properties. In this case, an automated segmentation procedure was performed based on the selection of the gray thresholds instead of some dots' layout topology or spatial disposition. A simple implementation of this technique allows obtaining an output of binary images, defined at two levels of gray: black or white, using a one-bit representation: 0 = black and 1 = white. This way, black pixels represent the background, while white pixels represent the object of interest. In addition, the range of gray levels that can be represented ranges from 0 to 255.

Threshold can be determined by one or more parameters. Intensity threshold uses only one parameter, and each pixel of the image is compared with the threshold, if the pixel intensity is greater (or lower), the pixel is set to 1; otherwise, it is set to 0. Typically, the region is segmented with a gray scale ranging from 50 to 255. If needed, morphological filters can be applied to improve the obtained contour definition.

After segmentation, the generated file presents anatomical parts and elements that do not belong to the face of the fetus. Therefore, it is needed to process it further with CAD software before creating the .STL model and the G-code for 3D printing.

**Figure 5:**
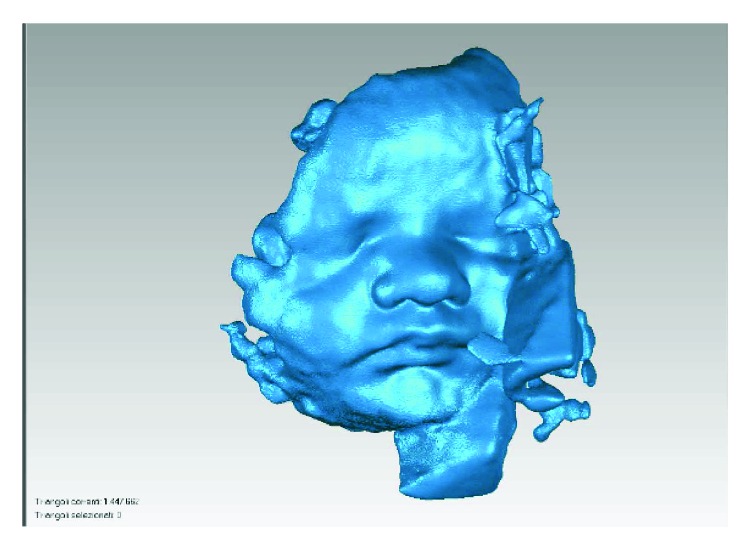
Example of a model obtained with Geomagic Studio.

Consequently, the face model is translated to a point cloud format (e.g., .xyz file format) and processed with Geomagic Studio software, (Figure 5), to clean and eliminate possible errors (holes, separate parts, etc.). Once the cloud is clean, the polygonal mesh surface can be built to generate a 3D CAD model. The obtained mesh is automatically and directly converted into an STL file format suitable for 3D printing. The total time amount usually needed to process a single model is between 5 and 6 hours.

**Figure 6:**
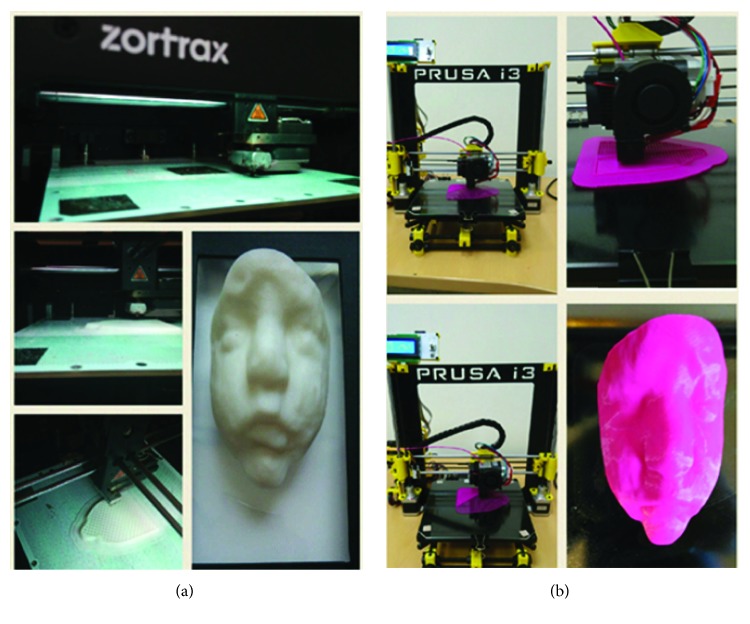
3D printed models of a 33-week-old fetus with the two different systems: (a) Zortax and (b) Prusa i3.

Two kinds of printers were used for printing the 3D models (Figure 6): a Zortrax M200 and a BQ Prusa i3 Hephestos. The Zortrax M200 is available at the CREAMI Lab (University of Naples—Federico II, Naples, Italy). It is based on FDM building technology and uses ABS materials. ABS produces a uniform and opaque finish of the printed models with pure color and excellent adhesion between the layers, ensuring easy removal of the supports. The Zortrax M200 has just one extruder and a single wire coil, which contributes to higher print quality and reliability, less dynamic masses, greater speed, and precision.

The BQ Prusa i3 Hephestos is also a FDM printer (available at the LAPI Lab of University of Cassino and Southern Lazio, Cassino, Italy) and uses PLA printing material which can also be used in home environments.  Table 1 summarizes the main printing parameters used for the present study.

The G-code file for printing is obtained using Cura software. The average time limit, to print a fetus face, is about 2.5 to 3 hours for both machines.

As a result, the proposed method is a valid approach to the construction of 3D fetal face models using only ultrasound images. The steps highlighted in our procedure are intended to provide a simple and repeatable process for reconstructing three-dimensional models that use images coming from routine pregnancy checks. In particular, this procedure allows to overcome the practical problems faced in the export and in the use of ultrasound images from most medical equipment used in diagnostic that do not allow the ability to reconstruct the model directly in some CAD format (such as for CT or MRI scans) or to easily export the entire stack of images acquired during the ultrasound examination.

Also, this method is valid when there is no need to reconstruct internal bone structures such as presented in [17–19] where, to better study the case of different fetal pathologies, authors recommend to use procedures supplementing information from ultrasound images with information coming from CT and/or MRI scans.

## 3. Survey Results and Discussion

This section presents the survey conducted, enquiring future parents about the possibility of using 3D baby face models for different purposes.

**Figure 7:**
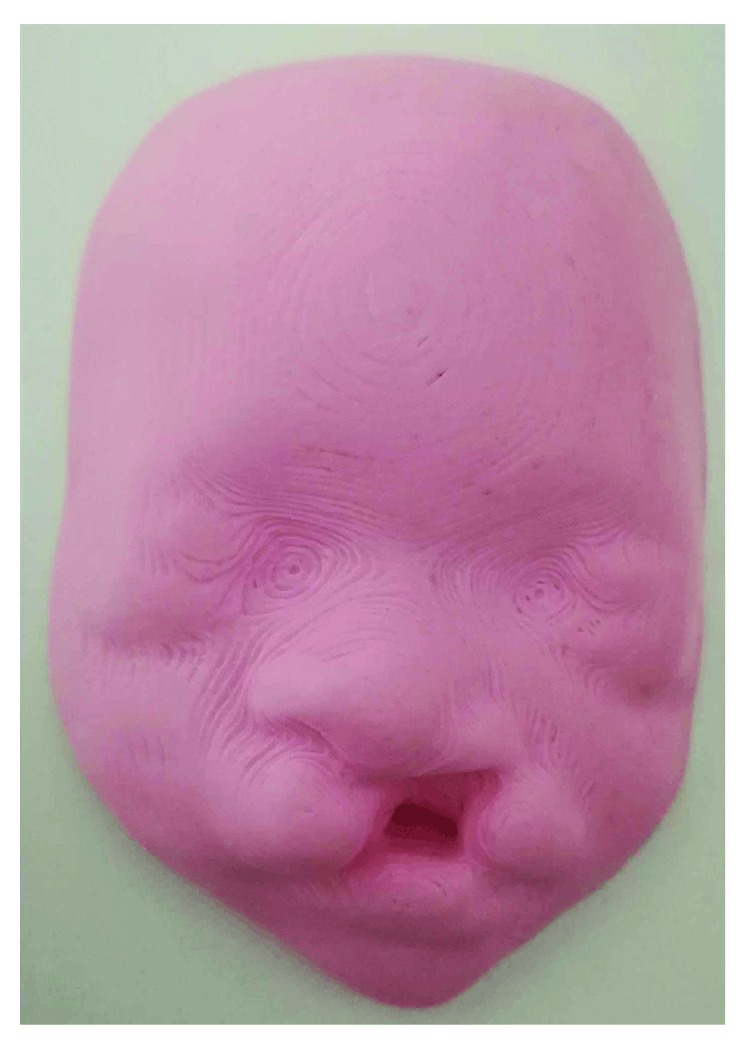
3D model of a 30-week-old fetus affected by a cleft lip pathology.

In fact, the aim of this survey is to investigate the use of this kind of 3D models from an emotional-relational point of view for the parents, and from a medical point of view, for the doctors to plan efficient surgical interventions on newborns and use 3D models as a support for parents in case of malformation. Also, the parents' perception of the model as a useful tool to promote the transition to parenthood was verified, especially in relation to possible facial malformations (Figure 7).

For the above-mentioned purposes and in order to perform a qualitative analysis, a questionnaire was created. It consisted of 26 questions subdivided into four different areas to collect different types of information: sociodemographic information; perception of emotions and tactile sensations evoked by the model, perception of model utility in case of malformation, and subjective evaluation of the model. 139 subjects were interviewed (83 females and 56 males), and data was collected at the Prenatal Diagnostic and Fetal Maternal Medicine Unit of Altamedica Clinic (Rome, Italy) from January to March 2017. The questionnaire was completed after viewing and touching the 3D face model of a 33-week-old fetus (Figure 6).

Table 2 reports the sociodemographic information (gender and age distribution) of the interviewed subjects.

Considering the level of instruction of the interviewed subjects, 52% of the whole sample has a university degree, while 45% possesses a high school degree, and only 3% has a lower secondary school qualification.  Table 3 reports the distribution of level of instruction considering mothers and fathers.

Considering the gestation period, the highest percentage, that is, around 27% of the 83 interviewed mothers, was in the third gestation month, followed by 22% in the fifth month and 17% in the sixth month and 11% in the eighth month. 45% of the interviewed parents declared they were not yet aware of the sex of their newborn, while 36% expected a female and 19%, a male baby. 63% of the respondents did not have any other children, while 36% were at the second pregnancy and only 1% is at the third pregnancy. Considering the familiarity of parents with new 3D printing technologies, 74% of the whole sample has heard something about 3D printing, while only 14% has heard about baby 3D printing.

Table 4 reports relevant questions regarding the perception of emotions and tactile sensations evoked by the 3D printed model and the values of the collected answers.

During the analysis of the collected answers, an additional evaluation of the impact of gender difference (mother or father) on the results was carried out. In Q11 referring to the subgroup of “fathers,” it can be seen that 70% of the 56 respondents answered positively. The same consideration may be applied to the subgroup “mothers” (83 persons), where the question encountered 73% positive answers. Also in Q12, a prevalence of positive answers was achieved by 65% of mothers and 57% of fathers.

Q13, Q14, and Q17 represent the most important questions within the questionnaire. They help understand whether the 3D print model can be perceived as an improvement of 3D/4D ultrasound images, giving parents the chance to discover the face of the child also through touch and not just through the images. For Q13, the same outcomes of the results are present for the two gender subgroups: 47% of mothers responded affirmatively, 25% responded negatively, and 28% were neutral; 57% of fathers answered affirmatively, 27% negatively, and 23% were neutral.

As to Q14, it encountered 27% of negative responses, 42% of positive, and the remaining 31% are neutral. In this case, a similar performance was observed for the mothers' subgroup, while a significant difference for the fathers' subgroup (39% of negative).

The results show that at this point, the 3D model does not increase or improve the parents' tactile perception of the newborn. Q17 does not highlight a prevalence of positive answers, it reports the 46% of positive answers (49% for mothers and 47% for fathers) and 38% of neutral answers followed by the 16% of negative answers. Since the average value (2.2 on 5) for usefulness of the model reached by the answers of Q16, it is assumed that at this stage, the model is not adequate to stimulate the creation of a stronger parent-child bond. Also, Q15 average value (2.5 on 5) for the usefulness of this model for transition to fatherhood did not reveal significant information. Considering average values of both subgroups, the answers are similar; especially in the case of fathers, it is evident that the model is considered insufficient. Q21 refers to the use of the 3D model as an aid in understanding the disease especially in case of facial deformities. The average value for the whole sample reached 3.5 on 5. Considering the different score levels attributed to this question by the entire sample, 48% of parents found the model “very useful” (score level 5); the same result was reported in both subgroups, that is, fathers and mothers. Considering Q21 answers in relation to the parents' level of education, it can be noted that the perceived utility increases hand in hand with the educational level. In fact, 22% of those who have a high school degree and 23% of those who have a university degree have considered the model “very useful” especially for the comprehension of facial malformations.

In the last section of the questionnaire, a subjective evaluation of the 3D model was studied. Questions Q20 and from Q22 to Q26 (see Table 4) are formulated to examine the value and meaning attributed by parents to the model (Q22, Q23, Q24, and Q25), its perception as a tool to enhance positive memories of pregnancy (Q20), and the individual features of the 3D model parents like (Q26). As to Q20, 44% of the whole sample considers the model as a tool to reinforce the positive memory of pregnancy. However, there is a slight percentage difference between the subgroups “fathers” and “mothers;” in fact, while 47% of “mothers” considers that the model would help to strengthen the positive memory of pregnancy, only 39% of “fathers” believes that the model serves this purpose. Q22 is concerned with the perceived value of the model. Compared to the whole sample, the average value is around 3.5. The same applies to subgroups “mothers” and “fathers” (see Table 4). Q24 and Q25 are related to the possibility of buying the 3D printed model. In this case, the sample responses highlight that only 27% of parents are willing to buy this model, and also they are not willing to pay large amounts of money (only a maximum of 20 euros/20 US$) for this kind of model.

On the other hand, Q23 and Q26 are two open questions. In particular, Q23 considers the possibility to own the 3D model (see Table 5), and Q26 gives an idea of what parents like about the model. For Q23, as reported in Table 5, the answers were grouped into some different choices: memory, birth announcement, create a bond with brothers/sisters, create a positive moment, and support in case of malformation. Since it was possible to select multiple answers, considering the whole sample, the choice with the strongest preference is memory (59%), followed by creating a positive moment (19%) and support in case of malformation with 16%. Analyzing the “father” and “mother” subgroup memory has the highest percentage (60% mothers and 57% fathers) of answers followed by the support in case of malformation (18% mothers and 13% fathers) and by creating a positive moment (16% mothers and 23% fathers).

In the case of Q26, the answers were grouped into five different categories: nothing, neutral, dimensions/size, utility/benefit, and sensation/emotions. Considering the whole sample, the category with the strongest preference is the fourth, related to model utility/benefit—with 35% (mostly in case of blind people)—followed by the third, dimensions/size—with 22%. Analyzing the “father” and “mother” subgroups, the fourth category has the highest percentage of answers followed by the third. Taking into account the educational level, again it was noticed that although the degree of study increases and the perception of the utility of the model increases, it has the opposite effect on the importance of the size. There is also a considerable percentage difference with regard to the first category (nothing), where university graduates have a higher percentage than high school graduates and therefore expresses a greater disinterest towards the model.

### 3.1. Results and Discussion

This section reports the considerations to analyze the different answers received and to evaluate the motivations of the presence of a significant amount of negative and neutral responses.

Since literature lacks studies considering the use of 3D printing models of fetuses for emotional and memory purposes, the analysis of the results, obtained with this preliminary survey, is fundamental also in comparison to some studies related to the use of 3D/4D ultrasound as a mean for strengthening the maternal-fetal bonding as in [22]. In particular, this study compares the effect of the third trimester 3D/4D ultrasound versus the 2D ultrasound of the fetal face considering a survey conducted with mothers to be and also a literature review. Its main results highlight that bonding increases after either a 3D/4D or 2D ultrasounds but it is stronger at better degrees of visibility and recognition.

Starting from these considerations, our survey was aimed to investigate how the use of 3D printed models of fetal faces (versus 2D, 3D/4D ultrasound images) may influence or improve the perceptions of the unborn babies by future parents both in relation to emotions and to specific medical purposes.

According to the opinions of the interviewed parents, the 3D model is considered a valuable tool to convey positive feelings related to the memory of pregnancy and a psychological support for the parents who are preparing to welcome their child as highlighted by Q11 and Q12.

However, other responses (Q13, Q14) did not show any clear positive response to the possibility of having a 3D physical face model, which was also related to the unwillingness to buy this model as an additional service to routine pregnancy analysis (Q24, Q25) or the poor perceiving of differences with 3D/4D images (Q17). Analyzing in a more detailed and nonaggregated way, the answers to question Q26 can go to read these neutral or negative responses such as the will not to “ruin,” the memory of the real moment of the birth of your child, the vision of the little for the first time leaving at that specific moment, and the possibility of touching it.

In addition, the possibility of having a 3D model of their newborn's face at hand seems more important not so much from an emotional point of view, but especially from a medical point of view, mainly in the presence of diseases or malformations. In fact, the 3D model allows parents to understand more clearly the extent of the disease the baby may have (e.g., cases of cleft lip or facial dysmorphism). Additionally, model utilization could also allow monitoring of pathology evolution and better planning of correctional surgery interventions. Medical staff also believe that using a detailed 3D model of the fetal face is useful in prenatal diagnosis, especially in cases of dysmorphism. In fact, 3D printed patterns contribute to improving prenatal evaluation in high-risk pregnancies and help doctors make timely decisions for treating the disease.

In fact, the 3D printed model contains all the anatomical features of the facial area involved and allows highlighting various bone structures of surgical interest. The model is particularly intuitive and very informative, and it allows surgeons to clearly understand the situation and plan carefully the access and maneuvering spaces. As a result, doctors can adopt optimal intervention strategy due to early diagnosis and avoid unnecessary surgical interventions. In addition, economic sustainability and, above all, reduction in costs associated with the programmed surgical operation should not be underestimated. In fact, knowing in advance the timing and dynamics of the surgery, surgeons will be prepared to perform the best taking into account both material and patient condition. All in all, the most important benefit of using a 3D model lies in the facilitation of the relationship between a physician and a patient, in this case between a physician and a parent, allowing better surgical planning.

According to the opinions of the interviewed parents, the 3D model is considered a valuable tool to convey positive feelings related to the memory of pregnancy and a psychological support for the parents who are preparing to welcome their child. The degree of positive interest in product models shows that 3D printing is the next step in 3D imaging especially in case of blind people as highlighted in answers of Q26.

## 4. Conclusions

In the present paper, the authors have analyzed the possible benefits of 3D printing in the use of 3D models for emotional/affective purposes and also for prenatal diagnosis of facial malformations. A method for the construction and 3D printing, using FDM technique of fetal faces from routine ultrasound images, emphasizing the most problematic steps and highlighting possible solutions, was introduced. Then, a questionnaire was designed and distributed to soon-to-be parents to evaluate the use of this type of model for emotional/affective purposes and to enhance the parents' understanding of prenatal diagnosis.

The results show that the 3D model can be considered a valuable tool to convey positive feelings related to pregnancy and, as a psychological support for the parents who are preparing to welcome their newborn children.

Thus, it can be concluded that 3D printing is gradually becoming one of the most interesting frontiers in the development of personalized medicine, which requires an interdisciplinary approach between the various skills, often located both inside the hospital and outside, for example, in universities, centers, or specialized companies. The maker plays the role of a mediator between the physician and the patient, thus enabling a more efficient evaluation of the clinical picture and an improvement in surgical planning. In addition, the creation of anatomical models could become an additional support for training and didactic activities. In fact, through the creation of 3D replicas in various materials, it would be possible to hold more precise consultations, both required by colleagues from other hospitals and patients themselves, in order to properly evaluate the situation and perform the necessary operations at a significantly reduced risk.

## Figures and Tables

**Table 1:** Printing parameters.

Parameters	Values/settings
Material	PLA, ABS
Quality: layer height	0.1–0.2 mm
Shell: wall thickness	1 mm
Infill	20%
Printing temperature	215°C (PLA)–255°C (ABS)
Diameter	1.75 mm
Flow	100%
Print speed	400 mm/s
Travel speed	120 mm/s
Build plate adhesion type	Raft
	Raft air gap 0.3 mm
	Initial layer Z overlap 0.15 mm
	Raft top layers 2

**Table 2:** Sociodemographic information of the parents' sample.

	Females		Males	
	Number	%	Number	%
*Interviewed subjects*				
Tot: 139	83	60	56	40
*Age distribution (years)*				
18–25	4	5	2	4
26–35	45	54	20	36
36–38	23	28	9	16
39–42	4	5	10	18
>42	7	8	15	27

**Table 3:** Level of instruction of the parents' sample.

Level of instruction	Mothers		Fathers	
	Number	%	Number	%
University degree	47	57	25	45
High school degree	33	40	30	54
Lower secondary school qualification	3	4	1	2

**Table 4:** Relevant questions concerning the perception of emotions and tactile sensations evoked by the 3D model.

Number	Question text	Kind of answer	Results		Gender subgroups	
			%	Average	Fathers %	Mothers %
Q11	What kind of feeling do you expect for the face pattern of a newborn baby to evoke?	Positive	72	—	70	73
		Negative	15	—	16	14.5
		Neutral	13	—	14	12.5

Q12	What kind of emotions do you feel touching the face pattern of a newborn baby?	Positive	62	—	57	65
		Negative	14	—	16	12
		Neutral	24	—	27	23

Q13	Would you like to be able to touch your child's face model even before her/his birth?	Yes	51	—	50	47
		No	26	—	27	25
		Neutral	26	—	23	28

Q14	Do you think the model would make your child's presence even more tangible?	Yes	42	—	34	47
		No	31	—	39	25
		Neutral	27	—	27	28

Q15	How would you rate the usefulness of this model for transition to fatherhood?	From 1 = not useful at all to 5 = very useful	—	2.5	Average	Average
					2.2	2.4

Q16	How would you rate the usefulness of this model in creating a stronger parental bond?	From 1 = not useful at all to 5 = very useful	—	2.3	Average	Average
					2.2	2.4

Q17	Do you perceive a difference between the 3D ultrasound and the 3D model?	Yes	46	—	41	49
		No	16	—	14	17
		Neutral	38	—	45	34

Q20	Do you think this model will help you to strengthen the positive memory of pregnancy?	Yes	44	—	39	47
		No	29	—	27	31
		Neutral	27	—	34	22

Q21	How would you evaluate the utility of this model in the event of face-to-face malformation?	From 1 = not useful at all to 5 = very useful	—	3.5	Average	Average
					3.4	3.5

Q22	What value/meaning would you attribute to this model?	From 1 = not useful at all to 5 = very useful	—	2.8	Average	Average
					2.7	2.8

Q23	Would you like to own this model for?	Open question	In the text			

Q24	Would you buy this model?	Yes	27		18	34
		No	39		41	37
		Neutral	34		41	29

Q25	How much would you be willing to spend for this model?	20 euros	68		73	65
		50 euros	20		13	25
		Neutral	12		14	10

Q26	What do you like most about the model?	Open question	In the text			

**Table 5:** Answers to Q23: would you like to own this model for?

Q23 Would you like to own this model for?		
Categories of responses	% mothers	% fathers
Memory	60	57
Birth announcement	10	4
Create a bond with brothers/sisters	1	2
Create a positive moment	16	23
Support in case of malformation	18	13
Not responding	1	7
